# METTL3 promotes the initiation and metastasis of ovarian cancer by inhibiting CCNG2 expression via promoting the maturation of pri-microRNA-1246

**DOI:** 10.1038/s41420-021-00600-2

**Published:** 2021-09-08

**Authors:** Xuehan Bi, Xiao Lv, Dajiang Liu, Hongtao Guo, Guang Yao, Lijuan Wang, Xiaolei Liang, Yongxiu Yang

**Affiliations:** 1 Department of Obstetrics and Gynecology, The First Hospital of Lanzhou University, Lanzhou, 730000 People’s Republic of China; 2 Department of Obstetrics and Gynecology, The First Hospital of Lanzhou University, Key Laboratory of Gynecologic Oncology Gansu Province, Lanzhou, 730000 People’s Republic of China

**Keywords:** Cancer, Cell biology

## Abstract

Ovarian cancer is a common gynecological malignant tumor with a high mortality rate and poor prognosis. There is inadequate knowledge of the molecular mechanisms underlying ovarian cancer. We examined the expression of methyltransferase-like 3 (METTL3) in tumor specimens using RT-qPCR, immunohistochemistry, and Western blot analysis, and tested the methylation of METTL3 by MSP. Levels of METTL3, miR-1246, pri-miR-1246 and CCNG2 were then analyzed and their effects on cell biological processes were also investigated, using in vivo assay to validate the in vitro findings. METTL3 showed hypomethylation and high expression in ovarian cancer tissues and cells. Hypomethylation of METTL3 was pronounced in ovarian cancer samples, which was negatively associated with patient survival. Decreased METTL3 inhibited the proliferation and migration of ovarian cancer cells and promoted apoptosis, while METTL3 overexpression exerted opposite effects. Mechanistically, METTL3 aggravated ovarian cancer by targeting miR-1246, while miR-1246 targeted and inhibited CCNG2 expression. High expression of METTL3 downregulated CCNG2, promoted the metabolism and growth of transplanted tumors in nude mice, and inhibited apoptosis. The current study highlights the promoting role of METTL3 in the development of ovarian cancer, and presents new targets for its treatment.

## Introduction

Ovarian cancer is a common malignant tumor in gynecology with a high mortality rate and poor prognosis, presenting a five-year survival rate of less than 45% [1]. Despite advances in therapeutic strategies, more than 239,000 new cases and 152,000 deaths were caused by ovarian cancer in 2018 globally [2]. The occurrence of ovarian cancer is subject to multiple internal and external factors, among which, multiple genetic dysfunctions may be directly involved in the tumorigenesis [3].

Investigations into epigenetics in ovarian cancer, including gene regulation by DNA methylation and histone modifications, hold promise for translation into improved clinical care [4]. DNA methylation, one of the most common and well-studied epigenetic modification, participates importantly in the regulation of gene expression [5]. The past few years have witnessed an unprecedented advance in tumor risk prediction, molecular typing, and drug combination therapy based on individual DNA methylation [6], following upon the discovery in 1974 of a methylated form of adenine group in mRNA called n6 methyladenosine (m6A) [7]. It came to be understood that m6A plays a critical role in RNA splicing, translation, and RNA stability, thus performing significant biological functions in cancer [8]. Methyltransferase-like 3 (METTL3) is a m6A methylase closely implicated in cancer biogenesis and development [9]. A previous study has shown that METTL3 is a main methyltransferase functioning in methylation processes [10], which is upregulated in bladder cancer and other malignant tumors in proportion to poor clinical prognosis [11, 12]. In ovarian cancer, METTL3 activity has been reported to accelerate cell proliferation and migration by regulating transcription of AXL receptor tyrosine kinase (AXL, referring to the Greek term “anexelekto”, which means uncontrolled) [13, 14]. Furthermore, METTL3 can catalyze the integration of the modified base m6A, and is responsible for various internal chemical modifications of noncoding RNAs [15, 16]. MicroRNAs (miRNAs) are small, noncoding RNAs of 18–22 nucleotides in length that play regulatory roles in the expression of mRNA selectively through complementary binding to the 3′-UTR of target mRNAs [17, 18]. Recently, an increasing number of researchers have demonstrated the importance of miR-1246 in cancers [19–21]. Interestingly, METTL3 can upregulate miR-1246 expression by identifying the m6A modification of pri-miR-1246 to promote the proliferation, migration, and invasion of colon cancer cells [20]. Accordingly, we used bioinformatics to detect the downstream target genes of miR-1246 in ovarian cancer, which showed that miR-1246 inhibited cyclin G2 CCNG2 expression by interacting with a binding site on the target molecule. Accumulating evidence has suggested that CCNG2 can suppress the progression of ovarian cancer [22–24]. However, no research has yet explored the effect of the METTL3/miR-1246/CCNG2 signaling axis in ovarian cancer.

In this study, we mainly analyzed the interaction among METTL3, miR-1246, and CCNG2 and identified their effects on progression in ovarian cancer. The present data revealed that METTL3 recognizes the m6A modification of pri-miR-1246, and then upregulates the expression of miR-1246, which inhibits CCNG2 expression, and thereby increases the ability of ovarian cancer cells to proliferate, migrate, and invade, while reducing the level of apoptosis, resulting in the promotion of ovarian cancer occurrence and development.

## Results

### METTL3 shows hypomethylation and high expression in ovarian cancer tissues and cells

**Fig. 1:**
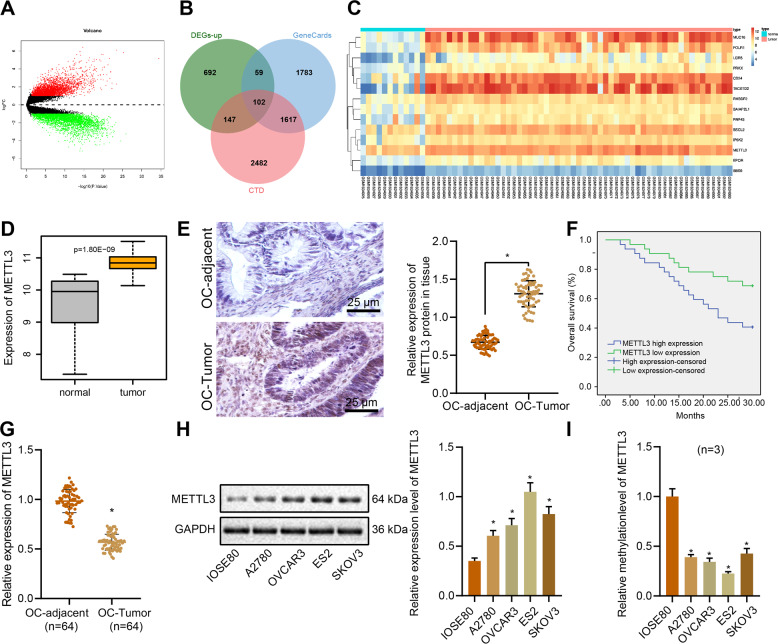
METTL3 gene shows hypomethylation and high expression in ovarian cancer tissues and cells. **A** The volcano graph of ovarian cancer-related gene expression from the GSE66957 microarray, where the red dots indicate high-expressed genes, the green dots indicate low-expressed genes, the abscissa indicates -log10 (p value), and the ordinate indicates log2FC value. **B** Venn diagram of the intersection of genes related to ovarian cancer in GeneCards database and CTD database and the top 1000 differentially expressed genes in ovarian cancer with the smallest *p* value. **C** Heat map of differential gene expression; the color scale from blue to orange indicates the expression value from small to large. **D** METTL3 is highly expressed in the GSE66957 microarray. **E** Immunohistochemistry examining METTL3 expression of ovarian cancer (*n* = 64) and adjacent normal tissues (*n* = 64) (400×). **F** According to the median relative expression of METTL3 detected by qPCR (the value is 1.785), patients were divided into high expression group and low-expression group; Kaplan–Meier analysis was used to investigate the relation between METTL3 expression and overall survival of patients. **G** METTL3 methylation was tested by MSP. **H** Western blot was used to detect the protein expression of METTL3 in IOSE80, A2780, OVCAR3, SKOV3, and ES2 cells. **I**, DNA methylation levels of METTL3 in human normal ovarian epithelial cell IOSE80 and four ovarian cancer cells (A2780, OVCAR3, SKOV3, and ES2) measured by MSP assay. *p < 0.05, compared with adjacent normal tissues or IOSE80 cells. The experiments were repeated three times with the most significant results presented.

Recent studies indicate that METTL3 expression correlates with ovarian carcinoma cellular proliferation and migration through the regulation of AXL transcription [13]. Through gene differential analysis of GSE66957, an ovarian cancer-related gene expression microarray, we obtained 7199 differentially expressed genes, of which 3599 genes were overexpressed and 3600 genes had low expression (Fig. 1A). A total of 3561 and 4379 ovarian cancer-related genes were obtained from the GeneCards database (https://www.genecards.org/, score ≥3.4) and CTD database (http://ctdbase.org/, score >35), respectively, and the intersection of the top 1000 ovarian cancer-related differentially expressed genes with the smallest p-value yielded 102 candidate genes (Fig. 1B). These candidate genes were imported into the GeneMANIA tool (http://genemania.org/) to obtain their co-expression relationship network (Supplementary Fig. 1A). In this analysis, 14 genes (BSCL2, BAIAP2L1, RHBDF2, IP6K2, MUC16, EPOR, RNF43, LGR5, BBS9, CD24, FOLR1, TACSTD2, METTL3, and PRKX) had high scores (score > 0.7) according to the website’s comprehensive score of gene coexpression relationship (Supplementary Table 1). Further, we plotted the differential expression heat map of these 14 genes in ovarian cancer (Fig. 1C). Previous work showed that the m6A methylase METTL3 is significantly upregulated in human liver cancer and other malignancies, and that its high expression predicts poor clinical prognosis [11]. METTL3 can promote the proliferation and migration of ovarian cancer cells by regulating the transcription of AXL [13]. Differential analysis of GSE66957, a relevant gene expression microarray for ovarian cancer, indicated that METTL3 was significantly highly expressed in ovarian cancer (Fig. 1D).

To parse out the role of METTL3 in ovarian carcinoma, we first detected the mRNA and protein expression of METTL3 in ovarian cancer tissues and adjacent normal tissues of 64 patients with ovarian cancer by RT-qPCR, immunohistochemistry, and Western blot analysis. The results showed that the mRNA and protein expression levels of METTL3 in ovarian cancer tissues were significantly increased compared with adjacent normal tissues (Fig. 1E, Supplementary Fig. 1B, C). Further, the Kaplan–Meier analysis showed that METTL3 high expression was associated with poor prognosis of ovarian cancer (Fig. 1F). Importantly, there was a distinct reduction in METTL3 methylation in the tumor samples, but no such was changed in paracancerous tissues (Fig. 1G), suggesting that extensive hypomethylation of METTL3 in ovarian cancer was negatively associated with the survival of patients with this carcinoma.

In further studies, we focused on METTL3 expression in normal ovarian epithelial IOSE80 cells and ovarian cancer cells, i.e., A2780, OVCAR3, SKOV3, and ES2. There was an upregulation of METTL3 in A2780, OVCAR3, SKOV3, and ES2 cells compared with IOSE80 cells (Fig. 1H, Supplementary Fig. 1D), among which the highest expression of METTL3 was in ES2 cells. METTL3 methylation in cancer cell lines was distinctly reduced, among which the ES2 cells showed the lowest METTL3 methylation (Fig. 1I) and were thus selected for subsequent analyses [25, 26].

### Decreased METTL3 expression inhibits the proliferation and migration of ovarian cancer cells and promotes apoptosis

**Fig. 2:**
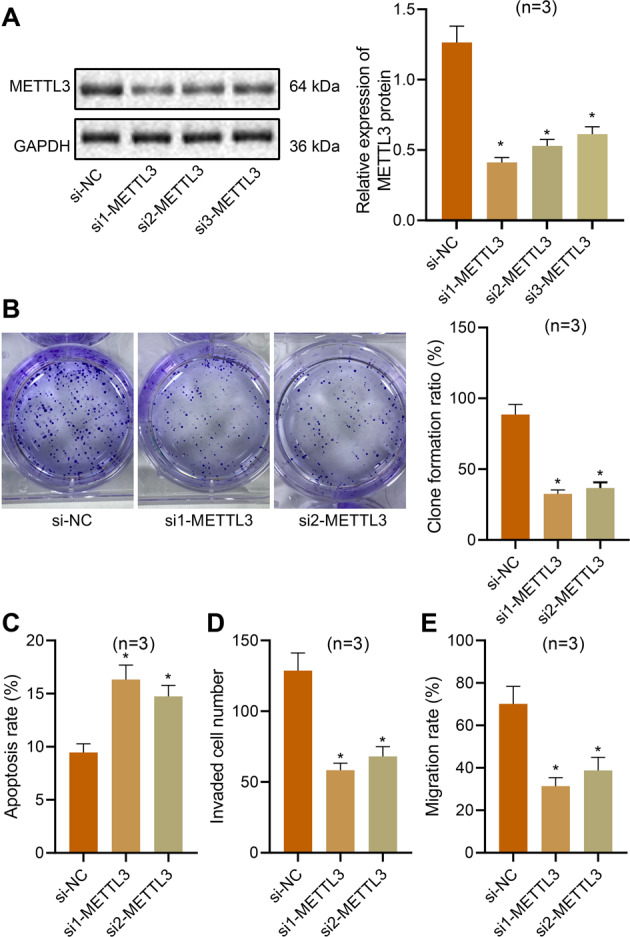
METTL3 silencing inhibits the proliferation, migration, and invasion of ES2 cells and promotes their apoptosis. **A** METTL3 protein expression in ES2 cells treated with si1-METTL3, si2-METTL3, or si3-METTL3 measured by Western blot. **B** Proliferation of ES2 cells treated with si2-METTL3 or si3-METTL3 by colony-formation test. **C** Flow cytometric analysis of apoptosis in ES2 cells treated with si2-METTL3 or si3-METTL3. **D** Invasion of ES2 cells treated with si2-METTL3 or si3-METTL3 measured by Transwell assay. **E** Migration of ES2 cells treated with si2-METTL3 or si3-METTL3 measured by scratch test. *p < 0.05, compared with the ES2 cells treated with si-NC. The experiments were repeated three times with the most significant results presented.

Next, we aimed to determine the effect of METTL3 on the proliferation, migration, invasion, and apoptosis of ovarian cancer cells. METTL3 mRNA and protein expression was decreased in ES2 and OVCAR3 cells treated with si1-METTL3, si2-METTL3, or si3-METTL3 (Fig. 2A, Supplementary Fig. 2A, B), among which si1-METTL3 and si2-METTL3 showed superior silencing efficiency and were selected for the subsequent experiments. Moreover, the proliferation (Fig. 2B, Supplementary Fig. 2C), migration (Fig. 2E, Supplementary Fig. 2F), and invasion (Fig. 2D, Supplementary Fig. 2E) of the si1-METTL3- or si2-METTL3-treated ES2 and OVCAR3 cells were reduced significantly, while the cell apoptosis was significantly increased (Fig. 2C, Supplementary Fig. 2D). These data suggested that silencing of METTL3 decreased ovarian cancer cell proliferation, migration, and invasion [27, 28].

### METTL3 overexpression promotes the proliferation, migration, and invasion, and inhibits apoptosis of ovarian cancer cells

**Fig. 3:**
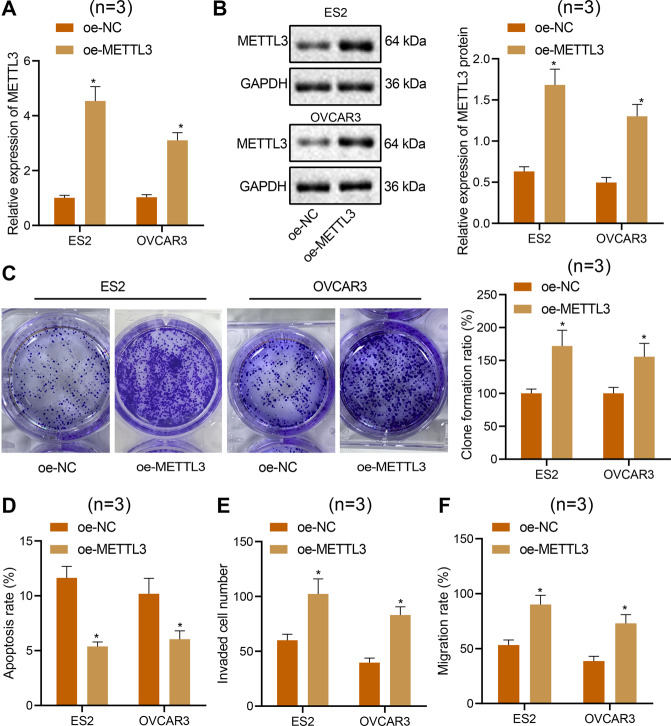
METTL3 overexpression promotes ES2 and OVCAR3 cell proliferation, migration, and invasion, while inhibiting apoptosis. **A** METTL3 expression in ES2 and OVCAR3 cells treated with oe-METTL3 tested by qPCR. **B** METTL3 protein expression in ES2 and OVCAR3 cells treated with oe-METTL3 tested by Western blot. **C** The proliferation of ES2 and OVCAR3 cells treated with oe-METTL3 detected by colony-formation assay. **D** Flow cytometric analysis of apoptosis of ES2 and OVCAR3 cells treated with oe-METTL3. **E** Invasion of ES2 and OVCAR3 cells treated with oe-METTL3 detected by Transwell assay. **F** Migration of ES2 and OVCAR3 cells treated with oe-METTL3 measured by scratch test. *p < 0.05, compared with the ES2 and OVCAR3 cells treated with oe-NC. The experiments were repeated three times with the most significant results presented.

Additionally, an oe-METTL3 plasmid was transfected into ES2, OVCAR3, and IOSE80 cells to investigate further the role of METTL3 in ovarian cancer. The results of RT-qPCR and Western blot analyses confirmed the overexpression efficiency of METTL3 in ES2, OVCAR3, and IOSE80 cells (Fig. 3A, B, Supplementary Fig. 3A, B). In addition, the proliferation (Fig. 3C, Supplementary Fig. 3C), migration (Fig. 3E), and invasion (Fig. 3F) of ES2, OVCAR3, and IOSE80 cells overexpressing METTL3 were significantly elevated, but the apoptosis (Fig. 3D, Supplementary Fig. 3D) was reduced. On the basis of these findings, we concluded that METTL3 overexpression promoted the proliferation, migration, and invasion of these cells, and reduced their apoptosis [27, 28].

### METTL3 aggravates ovarian cancer by targeting miR-1246

**Fig. 4:**
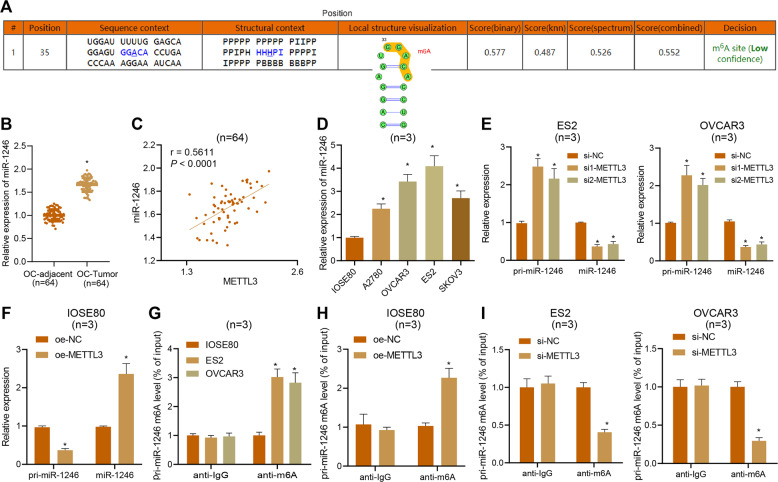
METTL3 promotes the maturation of pri-miR-1246 through m6A modification and upregulates the expression of miR-1246. **A** Possible m6A methylation modification sites in miR-1246. **B** Expression of miR-1246 in ovarian cancer (*n* = 64) and adjacent normal tissues (*n* = 64) assessed by qPCR. *p < 0.05, compared with adjacent normal tissues. **C** Correlation analysis of miR-1246 and METTL3 expression in human ovarian cancer tissues (*n* = 64). **D** The expression of miR-1246 in IOSE80, ES2, and OVCAR3 cells detected by qPCR. *p < 0.05, compared with IOSE80 cells. **E** The expression of pri-miR-1246 and miR-1246 in ES2 and OVCAR3 cells treated with si-METTL3 detected by qPCR. *p < 0.05, compared with ES2 and OVCAR3 cells treated with si-NC. **F** The expression of pri-miR-1246 and miR-1246 in IOSE80 cells treated with oe-METTL3 detected by qPCR. *p < 0.05 compared with IOSE80 cells treated with oe-NC. **G** m6A modification level of pri-miR-1246 in IOSE80, ES2, and OVCAR3 cells detected by Me-RIP. *p < 0.05, compared with IOSE80 cells. **H** m6A modification level of pri-miR-1246 in IOSE80 cells treated with oe-METTL3 detected by Me-RIP. *p < 0.05, compared with IOSE80 cells treated with oe-NC. **I**, m6A modification level of pri-miR-1246 in ES2 and OVCAR3 cells treated with si-METTL3 detected by Me-RIP. *p < 0.05, compared with ES2 and OVCAR3 cells treated with si-NC. The experiments were repeated three times with the most significant results presented.

METTL3 can recognize the m6A modification of pri-miR-1246 to upregulate miR-1246, and thereby promote the proliferation, migration, and invasion of colon cancer cells [20]. Therefore, our study focused on whether METTL3 played a role by the regulation of miR-1246 in ovarian cancer cells. The possible m6A methylation modification sites in miR-1246 were predicted via the SRAMP tool (Fig. 4A). Besides, RT-qPCR data revealed that miR-1246 was highly expressed in ovarian cancer tissues (Fig. 4B), and a correlation analysis demonstrated that the expression of miR-1246 was positively correlated with that of METTL3 (Fig. 4C). Moreover, we observed high expression of miR-1246 in ovarian cancer cells (Fig. 4D). Further, silencing METTL3 in ES2 and OVCAR3 cells or overexpression of METTL3 in IOSE80 cells showed that silencing of METTL3 augmented the expression of pri-miR-1246, while reducing that of miR-1246 (Fig. 4E). In contrast, overexpression of METTL3 resulted in a loss of pri-miR-1246 expression and an increase of miR-1246 expression, while silencing METTL3 led to opposite effects (Fig. 4F). These findings confirmed that METTL3 could promote the transformation of pri-miR-1246 into mature miR-1246 in ovarian cancer cells.

To verify further that METTL3 regulates the transformation of pri-miR-1246 into mature miR-1246 by its methyltransferase activity, we detected the methylation level of pri-miR-1246. The results from Me-RIP showed that the m6A modification level of pri-miR-1246 in ES2 and OVCAR3 cells was significantly increased when compared with IOSE80 cells (Fig. 4G); the m6A modification level of pri-miR-1246 was significantly increased after overexpression of METTL3 in IOSE80 cells (Fig. 4H), but significantly reduced following silencing of METTL3 in ES2 and OVCAR3 cells (Fig. 4I), suggesting that METTL3 mediated miR-1246 by the mechanism of m6A modification.

**Fig. 5:**
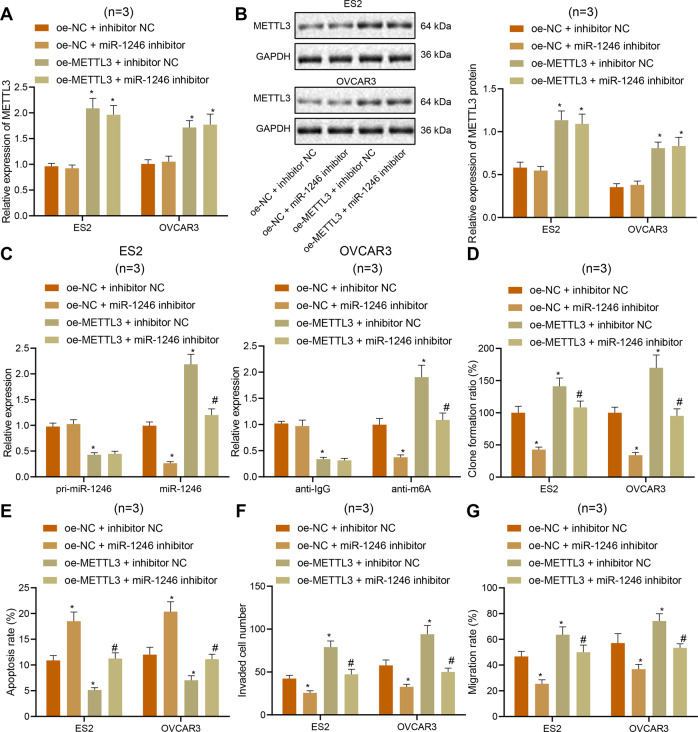
METTL3 induces proliferation, migration, and invasion of ovarian cancer cells while inhibiting their apoptosis via pri-miR-1246/miR-1246. ES2 and OVCAR3 cells were treated with miR-1246 inhibitor, oe-METTL3, or both. **A** Expression of METTL3 in ES2 and OVCAR3 cells detected by qPCR. **B** Protein expression of METTL3 in ES2 and OVCAR3 cells detected by Western blot. **C** The expression of pri-miR-1246 and miR-1246 in ES2 and OVCAR3 cells detected by qPCR. **D** Proliferation of ES2 and OVCAR3 cells measured by colony-formation assay. **E** Flow cytometric analysis of ES2 and OVCAR3 cell apoptosis. **F** Invasion of ES2 and OVCAR3 cells detected by Transwell assay. **G** Migration of ES2 and OVCAR3 cells detected by scratch test. *p < 0.05, compared with ES2 and OVCAR3 cells treated with oe-NC + inhibitor NC; ^#^*p* < 0.05, compared with ES2 and OVCAR3 cells treated with oe-METTL3 + inhibitor NC. The experiments were repeated three times with the most significant results presented.

Next, we simultaneously overexpressed METTL3 and inhibited miR-1246 in ES2 and OVCAR3 cells, and found that the mRNA and protein expression of METTL3 was significantly higher both inES2 cells and OVCAR3 cells treated with oe-METTL3 + inhibitor NC (Fig. 5A, B), in which the expression of pri-miR-1246 was significantly lower, and miR-1246 expression was significantly higher (Fig. 5C). Furthermore, the proliferation, migration, and invasion of ES2 cells and OVCAR3 cells were significantly increased, and their apoptosis level was significantly decreased (Fig. 5D–G). Compared with treatment with oe-NC + inhibitor NC, the miR-1246 expression was significantly lower in ES2 cells and OVCAR3 cells treated with oe-NC + miR-1246 inhibitor (Fig. 5C); the proliferation, migration, and invasion of ES2 cells and OVCAR3 cells were significantly decreased, and their apoptosis level was significantly increased (Fig. 5D–G) [29–31]. Collectively, we show that METTL3 can promote the maturation of pri-miR-1246 and upregulate the expression of miR-1246 through m6A modification, thereby promoting proliferation, migration, and invasion of ovarian cancer cells while inhibiting their apoptosis.

### miR-1246 targets CCNG2 in ovarian cancer cells

**Fig. 6:**
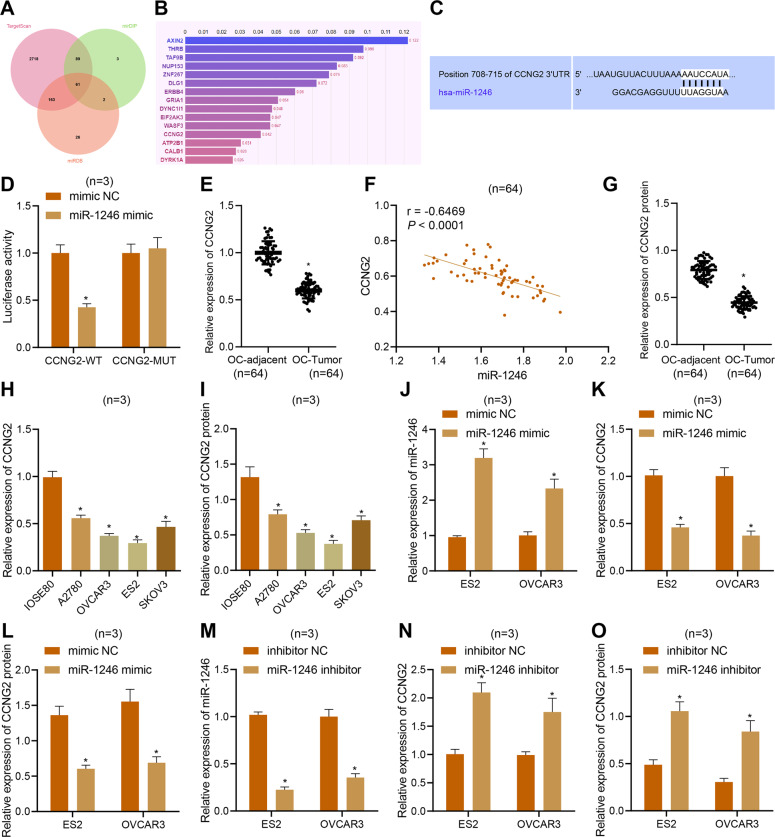
miR-1246 targets CCNG2 and reduces its expression in ovarian cancer cells. **A** Venn diagram of the intersection of the potential target genes of miR-1246 predicted by miRNA target gene prediction tools TargetScan, mirDIP, and miRDB. **B** Bar graph of correlation scores between candidate target genes and ovarian cancer. **C** Bioinformatics predicted that miR-1246 targeted CCNG2 and predicted its binding sites. **D** The dual-luciferase reported assay examined whether miR-1246 bound to CCNG2 mRNA. E, The mRNA levels of CCNG2 in human ovarian cancer tissues (*n* = 64) and adjacent normal tissues (*n* = 64) detected by qPCR. **F** Correlation analysis of miR-1246 and CCNG2 mRNA expression in human ovarian cancer tissues (*n* = 64). **G** Western blot analysis of CCNG2 protein expression in human ovarian cancer tissues (*n* = 64) and adjacent normal tissues (*n* = 64). **H**, **I** qPCR and Western blot were used to detect CCNG2 expression in IOSE80, A2780, OVCAR3, SKOV3, and ES2 cells. **J** The expression of miR-1246 in the ES2 and OVCAR3 cells treated with miR-1246 mimic detected by qPCR. **K**, **L** The expression of CCNG2 in ES2 and OVCAR3 cells treated with miR-1246 mimic examined by qPCR and Western blot, respectively. **M** Expression of miR-1246 in ES2 and OVCAR3 cells treated with miR-1246 inhibitor detected by qPCR. **N**, **O** CCNG2 expression in ES2 and OVCAR3 cells treated with miR-1246 inhibitor detected by qPCR and Western blot, respectively. In panel **D**, **J**–**L**, *p < 0.05, compared with mimic NC. In panels **E** and **G**, *p < 0.05, compared with adjacent normal tissues. In panels **H**, **I**, *p < 0.05, compared with IOSE80 cells. In panels **M**–**O**, *p < 0.05, compared with inhibitor NC. The experiments were repeated three times.

In order to explore further the mechanism by which miR-1246 affects the proliferation, migration, invasion, and apoptosis of ovarian cancer cells, we next used miRNA target gene prediction tools TargetScan, mirDIP, and miRDB to predict 3031, 155, and 252 potential target genes of miR-1246, respectively. After intersection, 61 candidate target genes were obtained (Fig. 6A). The candidate target genes were introduced into Phenolyzer to obtain a bar graph of the correlation between genes and ovarian cancer (Fig. 6B), which showed that 15 genes had higher correlation scores with ovarian cancer. Further review of related literature indicated that CCNG2 was weakly expressed in ovarian cancer [24]. We first predicted through the bioinformatics website TargetScan that miR-1246 can target the 3′-UTR of CCNG2 (Fig. 6C). The results of dual-luciferase reporter gene assay further confirmed that the luciferase activity of the miR-1246 mimic and CCNG2-WT plasmid cotransfection group was significantly lower than that of the mimic NC and CCNG2-WT cotransfection group, indicating that miR-1246 can indeed target and inhibit CCNG2 activity (Fig. 6D). Expression determination by RT-qPCR and Western blot showed that CCNG2 expression was decreased in ovarian cancer tissues and cells, whereas the opposite effects were seen with miR-1246 (Fig. 6E–I). From the above results, we hypothesized that miR-1246 plays a role in targeting CCNG2 in ovarian cancer. Moreover, CCNG2 expression was decreased by miR-1246 mimic and increased by miR-1246 inhibitor (Fig. 6J–O). These findings suggested that miR-1246 targeted and inhibited CCNG2 expression in ovarian cancer cells [32].

### miR-1246 enhances proliferation, migration, and invasion of ovarian cancer cells by reducing CCNG2

**Fig. 7:**
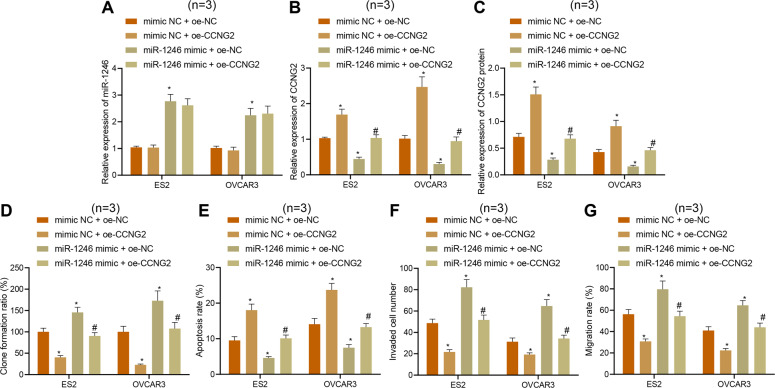
miR-1246 stimulates proliferation, migration and invasion of ES2 and OVCAR3 cells by targeting CCNG2. ES2 and OVCAR3 cells were treated with miR-1246 mimic, oe-CCNG2, or both. **A** The expression of miR-1246 in ES2 and OVCAR3 cells. **B**, **C** The expression of CCNG2 in ES2 and OVCAR3 cells examined by qPCR and Western blot. **D** Proliferation of ES2 and OVCAR3 cells measured by colony-formation assay. **E** The apoptosis in ES2 and OVCAR3 cells measured by flow cytometry. **F** The invasion of ES2 and OVCAR3 cells measured by Transwell assay. **G** The migration of ES2 and OVCAR3 cells measured by scratch test, *p < 0.05, compared with ES2 and OVCAR3 cells that were treated with mimic NC + oe-NC. ^#^*p* < 0.05, compared with ES2 and OVCAR3 cells that were treated with miR-1246 mimic + oe-NC. The experiments were repeated three times with the most significant results presented.

To verify whether miR-1246 promotes ovarian cancer cell proliferation, invasion, and migration through targeting CCNG2 expression, miR-1246 and CCNG2 were simultaneously overexpressed in ES2 and OVCAR3 cells. The results showed that compared with the treatment with mimic NC + oe-NC, there were no significant differences in miR-1246 expression, but elevated CCNG2 expression was observed in the ES2 and OVCAR3 cells treated with mimic NC + oe-CCNG2, accompanied with inhibited proliferation, migration, and invasion, as well as enhanced apoptosis (Fig. 7A–G). However, treatment with miR-1246 mimic + oe-NC increased miR-1246 expression but reduced CCNG2 expression accompanied with enhanced proliferation, migration, and invasion, as well as decreased apoptosis (Fig. 7A–G). Compared with the treatment with miR-1246 mimic + oe-NC, there was no significant difference in miR-1246 expression, but elevated CCNG2 expression was observed in the presence of miR-1246 mimic + oe-CCNG2, accompanied with inhibited proliferation, migration, and invasion, as well as enhanced apoptosis (Fig. 7A–G) [29–31]. Simultaneous silencing of miR-1246 and CCNG2 in IOSE80 cells reversed the promoting effect of miR-1246 on the proliferation of ovarian cancer cells (Supplementary Fig. 4). The above findings supported the claim that miR-1246 enhanced proliferation, migration, and invasion of ovarian cancer cells by targeting CCNG2.

### Overexpression of METTL3 promotes the tumorigenic ability of ovarian cancer cells in vivo through the miR-1246/CCNG2 axis

**Fig. 8:**
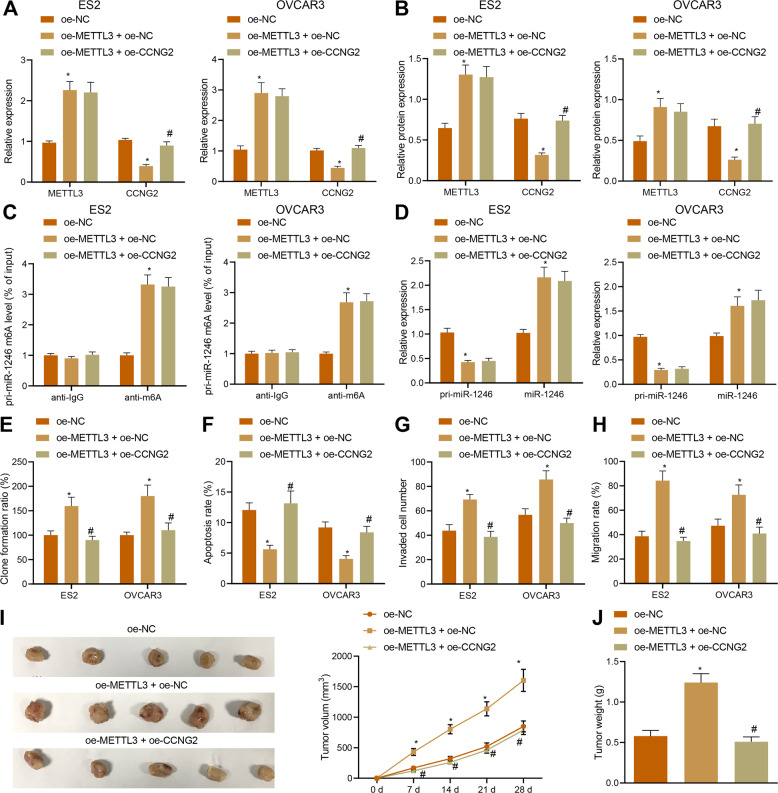
METTL3 induces the proliferation, invasion, and migration in vitro, as well as tumor formation in vivo of ovarian cancer cells by regulating the miR-1246/CCNG2 axis. ES2 and OVCAR3 cells were treated with oe-METTL3, oe-CCNG2, or both. **A**, **B** Expression of METTL3 and CCNG2 in ES2 and OVCAR3 cells detected by qPCR and Western blot. **C** The m6A modification levels of pri-miR-1246 in ES2 and OVCAR3 cells detected by Me-RIP assays. **D** The expression of pri-miR-1246 and miR-1246 in ES2 and OVCAR3 cells detected by qPCR. **E** The proliferation of ES2 and OVCAR3 cells detected by colony-formation assay. **F** The apoptosis of ES2 and OVCAR3 cells detected by flow cytometry. **G** Invasion of ES2 and OVCAR3 cells detected by Transwell assay. **H** The migration of ES2 and OVCAR3 cells detected by scratch test. Nude mice were injected with ES2 and OVCAR3 cells treated with oe-METTL3, oe-CCNG2, or both. **I** Representative images showing xenografts in nude mice and the tumor volume measured every seven days within four weeks. *n* = 12. **J** Tumor weight of nude mice. *n* = 12, *p < 0.05, compared with ES2 and OVCAR3 cells or mice treated with oe-NC. ^#^*p* < 0.05, compared with ES2 and OVCAR3 cells or mice treated with oe-METTL3 + oe-NC. The experiments were repeated three times.

**Fig. 9:**
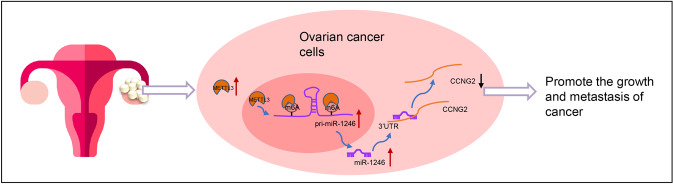
Schematic map of the function of METTL3 in ovarian cancer. METTL3 can recognize the m6A modification of pri-miR-1246, thus up-regulating the expression of miR-1246, which inhibited CCNG2 to accelerate cell proliferation, migration, and invasion, but suppresses apoptosis of ovarian cancer cells, promoting the carcinogenesis and processing of ovarian cancer.

Finally, we shifted our attention to determine whether METTL3 affects the proliferation, migration, and invasion of ovarian cancer cells through the miR-1246/CCNG2 axis. Overexpression of METTL3 in ES2 and OVCAR3 cells increased the m6A level of pri-miR-1246 and miR-1246 expression while decreasing pri-miR-1246 and CCNG2 expression (Figs. 8A–D, 9), in addition to provoking an increase in the proliferation, migration, and invasion of ES2 and OVCAR3 cells, and a decrease in apoptosis (Fig. 8E–H). However, the addition of oe-CCNG2 exerted no effects on the METTL3 expression, m6A level of pri-miR-1246, miR-1246 expression, and pri-miR-1246 expression, but instead elevated CCNG2 expression (Fig. 8A, B) accompanied by inhibited proliferation, migration, and invasion of ES2 and OVCAR3 cells, and enhanced their apoptosis (Fig. 8E–H). At the same time, the tumorigenesis ability of nude mice increased significantly after oe-METTL3 treatment, but the addition of oe-CCNG2 exerted the opposite effect (Fig. 8I–K). These results indicated that overexpressed METTL3 enhanced the tumorigenic ability of ovarian cancer cells in vivo via the miR-1246/CCNG2 axis [33].

## Discussion

Epigenetic regulatory factors mainly comprise post-translational modified cytokines, histone modifications, and noncoding RNAs [34], which are able to regulate genome and environment interactions, but do not change the underlying DNA sequence [35]. DNA methylation, as the most common epigenetic modification, is biologically necessary to silence retroviral elements that account for 5–8% of the human genome [36]. Understanding how the DNA methylation affects ovarian cancer development will help to propose more effective treatments. Our study demonstrated that the expression and methylation level of METTL3 has a tight relationship with ovarian cancer development and further identified that METTL3 stimulated the m6A modification of miR-1246 to promote miR-1246 expression, which subsequently downregulates the expression of CCNG2 (Fig. 9).

Our results showed that the METTL3 genome sequence was hypomethylated in ovarian tumor samples and cells, indicating that a high level of METTL3 correlated with poor prognosis of patients with ovarian cancer, and suggesting that METTL3 might participate in ovarian cancer development. It is known that METTL3 participates in all stages of the RNA life cycle and affects tumor formation by regulating m6A modifications of key oncogenes or tumor suppressor genes [12]. Recent evidence has shown significantly upregulated METTL3 expression in various human malignancies [20] and its high expression is tightly associated with poor clinical prognosis [11]. METTL3 was previously reported as a tumor-promoting factor in ovarian cancer [13], in accordance with our present results that overexpression of METTL3 could accelerate proliferation, migration, and invasion, while inhibiting apoptosis of ovarian cancer cells.

Subsequently, we analyzed the mechanisms whereby METTL3 might regulate miR-1246 [20]; our results showed that METTL3 could identify the m6A modification of pri-miR-1246, and further promoted the maturation of pri-miR-1246. At the RNA level, more than 100 types of post-transcriptional modifications have been reported, among which one of the most common is the m6A RNA methylation [37]. m6A modification involves in a series of downstream functions, including miRNA processing, nuclear export, translation modulation, and RNA degradation [8]. Coincidentally, perhaps, METTL3 is reported to catalyze the deposition of m6A and is responsible for abundant internal chemical modification of noncoding RNAs [15, 16]. METLL3 is considered to promote the m6A-circRNA formation in a manner distinct from the m6A modifications of linear mRNAs [38]. Specially, it has been reported that the maturation of miRNAs, such as let-7e, miR126, miR25, miR93, miR4485, and miR335, could be promoted through DGCR8 processing after being marked for recognition by METTL3 [39]. These findings suggest that METLL3 acts as a key post-transcriptional modification in normal human physiological process or diseases by regulating the m6A modification of noncoding RNAs. In recent years, an increasing number of researchers have demonstrated high expression of miR-1246 in samples from patients with ovarian cancer, and this revealed the importance of miR-1246 as a promising clinical diagnostic biomarker or therapeutic target in ovarian cancer [19, 40, 41]. More importantly, it was reported that METTL3 can promote colorectal cancer metastasis through methylating pri-miR-1246 and accelerating its maturation [20]. METTL3 may promote the maturation of pri-miR-1246 in a DGCR8-dependent manner [39]. Consistent with those findings, our data provide evidence that METTL3 could play a tumor-promotive role in ovarian cancer through regulating the maturation of miR-1246.

An important miR-1246-mediated mechanism was further highlighted in this study, in showing that miR-1246 could target CCNG2 through binding to CCNG2 3′-UTR. Previous research indicated that miR-1246 interacted with CCNG2 expression in different human cancers. For instance, miR-1246 could promote proliferation, invasion, and chemoresistance of breast cancer cells by targeting CCNG2 [42]. miR-1246 expression functioned importantly in chemoresistance and stemness in pancreatic cancer through interacting with CCNG2 [43]. miR-1246 has also been found to enhance CCNG2-mediated cancer stemness and drug resistance in oral carcinomas [44]. Encoded by the CCNG2 gene, CCNG2 is a protein belonging to a group of unconventional cyclins that function in maintaining cellular quiescence and cell cycle arrest [45]. Accumulating evidence showed that CCNG2 expression has an inverse association with the development of various cancers, such as breast [46], lung [47], colorectal [48], and gastric cancers [49]. It has been widely observed that CCNG2 expression has a positive correlation with patient survival [50]. CCNG2 is highly unstable and can reduce cell proliferation in ovarian cancer, conceivably by inhibiting epithelial-to-mesenchymal transition, cell migration, and invasion [24]. The results our our gain-of-function and rescue experiments in vitro substantiated that downregulation of CCNG2 was responsible for the pro-proliferative and anti-apoptotic effects of miR-1246.

Taken together, our results provide evidence to support the proposition that METTL3 plays a carcinogenic role in ovarian cancer. METTL3 can recognize the m6A modification of pri-miR-1246, and thus upregulate the expression of miR-1246, which inhibited CCNG2 to accelerate cell proliferation, migration, and invasion, but suppressed apoptosis of ovarian cancer cells, with the net effect of promoting the progression of ovarian cancer. These findings shed new light on the molecular mechanisms underlying the occurrence and development of ovarian cancer. Future studies will explore the possibility of regulating the METTL3/miR-1246/CCNG2 axis as targeted therapies for ovarian cancer.

## Materials and methods

### Ethics statement

The current study was approved by the Ethics Committee of the First Hospital of Lanzhou University and performed in strict accordance with the *Declaration of Helsinki*. All patients and their families signed informed consent documentation before sample collection. Animal experiments were performed with the approval of Animal Ethics Committee of the First Hospital of Lanzhou University and strictly following the Guide for the Care and Use of Laboratory Animals published by the US National Institutes of Health. Extensive efforts were made to ensure minimal suffering of the included animals.

### Clinical sample collection

Patients (*n* = 64, aged 20–67 years) with ovarian cancer who underwent cancer resection at the First Hospital of Lanzhou University were selected from January 2014 to January 2016. Patients had not received radiotherapy, chemotherapy, or immunotherapy before the operation. All surgical specimens were taken from the nonnecrotic bleeding area of the cancer tissue center and the adjacent normal tissues (resected at least 1 cm outside the tumor lesion boundary and pathologically confirmed to be nontumor tissues), which were confirmed by the pathological tests. Samples were stored in a −80 °C refrigerator for further experiments. The follow-up started from the end of the operation and ended in June 2018, which lasted for 3–30 months. The Kaplan–Meier method was used to analyze the relationship between the expression of METTL3 and the overall survival of patients with ovarian cancer. The clinicopathological features of patients are displayed in Supplementary Table 2.

### Detection of DNA methylation level by MSP

The total DNA of ovarian cancer tissues or cells was extracted by DNAzol kit (Invitrogen, Carlsbad, CA, USA) [51, 52]. The total DNA was treated by the EZ DNA Methylation–Gold Kit (Zymo Research, Irvine, CA, USA) for sodium bisulfite modification. After this treatment, the specimens were kept in the refrigerator at −20 °C until analysis. The following METTL3 methylation primers were designed: methylated (M)-METTL3-Forward: 5′-GAGGGCGGATTACGAGGTGAGGAGT-3′,

M-METTL3-Reverse: 5′-TCCTACCGAACCTCCCGAATAACT-3′; and unmethylated primer U-METTL3-Forward: 5′-GAGGGTGGATTATGAGGTGAGGAGT-3′,

U-METTL3-Reverse: 5′-TCCTACCAAACCTCCCAAATAACT-3′. The reaction conditions were as follows: predenaturation at 95 °C for 10 min, followed by 30 cycles of denaturation at 95 °C for 1 min, annealing at 60 °C for 30 s, extension at 72 °C for 1 min, final extension at 72 °C for 5 min, and storage at 4 °C. The amplified products were separated by 2% agarose gel electrophoresis and analyzed by ChemiDoc XRS + (Bio-Rad, Hercules, CA, USA).

### Reverse-transcription quantitative polymerase chain reaction (RT-qPCR)

Total RNA was extracted from cells and tissues by TRIzol reagents (Invitrogen, Carlsbad, CA, USA) with its concentration and purity detected by a Nanodrop2000 microultraviolet spectrophotometer (1011U, Nanodrop, Wilmington, DE, USA). The extracted RNA was reversely transcribed to cDNA according to the instructions of PrimeScript RT Kit (RR047A, Takara, Japan), and microRNAs were detected according to TaqMan MicroRNA Assays Reverse Transcription primer (4427975, Applied Biosystems, USA). Primers for METTL3, miR-1246, pri-miR-1246, and CCNG2 were designed and synthesized by Takara (Supplementary Table 3). ABI 7500 quantitative PCR instrument was used for real-time fluorescence quantitative PCR detection. GAPDH and U6 served as internal controls. The relative transcription level of the target gene was calculated by the 2^−△△CT^ method. Each sample was analyzed in triplicate [53].

### Immunohistochemistry (ICH)

The paraffin-embedded 5 μM sections of human ovarian cancer and adjacent normal tissues were prepared, conventionally dewaxed, and hydrated. After antigen retrieval using a microwave, the section was blocked by 1% BSA solution for 1 h at room temperature and incubated with METTL3 primary antibody (rabbit, 1:500, Abcam, Cambridge, UK) overnight at 4 °C. After PBS washing, the section was then incubated with HRP-labeled IgG (Boster, Wuhan, China) at room temperature for 1 h, followed by DAB staining (Boster). Finally, the sections were stained in hematoxylin (Servicebio, Wuhan, China), dehydrated by gradient alcohol, and observed under microscopy after sealing.

### Western blot analysis

The tissues and cells of each group were lysed in RIPA solution (Beyotime, Shanghai, China) for 30 min and centrifuged at 14000 RPM and 4 °C, and the supernatant collected. The protein concentration was determined by the BCA method (Pierce, Rockford, IL, USA). The proteins were separated in 4% or 10% concentrated gel and transferred to a PVDF membrane. The PVDF membrane was blocked by placement in 5% skimmed milk powder at room temperature for 1 h. Next, the membranes were incubated at 4 °C with rabbit anti-METTL3 (1:1000, Abcam) or rabbit anti-CCNG2 (2.5 µg/mL, Sigma, St. Louis, MO, USA) overnight. After washing with PBST, the membranes were incubated with goat anti-rabbit IgG labeled with horseradish peroxidase (HRP) (1:1000, Santa Cruz, Dallas, TX, USA) at room temperature for 1 h. The blots were visualized by enhanced chemiluminescence (ECL) reagents (Thermo Fisher). The images were observed and captured using the ChemiDoc Imaging System (Bio-Rad, Hercules, CA, USA). GAPDH (Rabbit anti-GAPDH, 1:100–1:1000, Santa Cruz) was used as an internal reference, and the images were analyzed by ImageJ software [22].

### Cell culture and transfection

Four ovarian cancer cell lines, including OVCAR3 and SKOV3 purchased from ATCC (Manassas, VA, USA), as well as A2780 and ES2 purchased from Procell Life Science & Technology Co., Ltd. (Wuhan, China) (stored at European Collection of Authenticated Cell Culture, ECACC), and human normal ovarian epithelial cell line IOSE80 (Oulu Biotech., China) were used [54–56]. Cells were cultured in RPMI-1640 (Gibco, Carlsbad, CA, USA) containing 10% fetal bovine serum (FBS) (Gibco), 100 μg/mL streptomycin, and 100 U/mL penicillin in a 5% CO_2_ incubator (Thermo Fisher) at 37 °C. After 24 h of culture, the cells with confluence of about 75% were transiently transfected with oe-METTL3, si-METTL3 (si1-METTL3, si2-METTL3, and si3-METTL3), miR-1246 inhibitor, miR-1246 mimic, miR-1246 inhibitor, and oe-CCNG2, as well as their negative controls according to the instructions of the Lipofectamine 2000 kit (Invitrogen, Carlsbad, CA, USA). The sequences for si-NC, si-METTL3-1, si-METTL3-2, and si-METTL3-3 are shown in Supplementary Table 4. Plasmids, siRNA mimic, and inhibitor were obtained from Sino Biological (Beijing, China). After 6 h of transfection, the culture was replaced by fresh medium. Cells were collected after further culturing for 48 h.

### Colony-formation assay

Ovarian cancer cells at the logarithmic growth phase were collected, digested by 0.25% trypsin, triturated, counted, and adjusted to 1 × 10^6^ cells/mL. Each group of cells was inoculated with a gradient density of 50, 100, and 200 cells, respectively, in a 10-cm dish that was cultured in a 37 °C 5% CO_2_ incubator for 2–3 weeks. The culture was terminated when the clone was visible in the culture dish. Then, the cells were fixed by 4% paraformaldehyde (5 mL, Invitrogen) for 15 min and stained by Giemsa staining solution (5 mL, Invitrogen) for 10–30 min. Finally, the cells were washed slowly with PBS and dried in air. The plate was observed under the inverted microscope (DMi8-M, Leica, Wetzlar, Germany). The number of cell clones was recorded and the clone-formation rate was calculated as the number of formed clones / the number of inoculated cells [57, 58].

### Flow cytometry assay

Cell apoptosis was detected by Annexin V-FITC/PI double staining according to previous studies [59]. Ovarian cancer cells were seeded into a 6-well plate at a density of 2 × 10^5^ cells/well, which were transfected at the concentration of 100 nM. After 72 h of transfection, the culture solution was removed, washed once with PBS, and digested with trypsin. Cells were collected in a 15-mL centrifuge tube and the supernatant was discarded after centrifugation at 800 g. According to the instructions of Apoptosis Detection Kit (BD Bioscience, San Jose, CA, USA), the cells were resuspended in 500 μL of binding buffer, and then 5 μL of FITC and 5 μL of PI were added into the buffer for incubation with cells for 15 min. After that, the cell apoptosis was detected by FACSCalibur (BD Bioscience).

### Transwell experiment

Ovarian cancer cells were resuspended in the Opti-MEMI (Invitrogen, USA) containing 10% BSA (Sigma) with the cell density adjusted to 3 × 10^4^ cells/mL. The migration/invasion experiments were performed by a 24-well plate of 8-μm Transwell chamber (Corning, NY, USA). The cell suspension (100 μL) was added to the upper chamber of each well, while 10% RPMI1640 medium (600 μL) was added to the lower chamber of the well. Then the cells were incubated at 37°C and 5% CO_2_. In the migration test, the cells were fixed with 4% paraformaldehyde for 30 min after 48 h of incubation, then incubated with 0.2% Triton X-100 (Sigma) solution for 15 min, and dyed with 0.05% gentian violet for 5 min. In the invasion experiment, 50 μL of Matrigel (Sigma) was added to the chamber 48 h before the cells were fixed and stained. The numbers of stained cells were counted under a microscope (DMi8-M, Leica). Five fields of view were randomly selected for counting, and the average number of cells was recorded. The experiment was repeated three times [60].

### Wound-healing test

After 48 h of transfection, ovarian cancer cells were seeded into a 6-well plate at a density of 5 × 10^5^ cells/well. When completely adhered to the wall, the cells were scratched in 2-mm width in the middle of each well and further cultured for 24 h. Photos were taken at 0 and 24 h after scratching, and the rate of scratch healing was calculated by Image-Pro Plus 6.0. Each group was performed at least three biological repeats [27].

### Dual-luciferase reporter assay

The predicted region of CCNG2 mRNA 3′-UTR as well as binding-site fragments and mutant fragments of miR-1246 were inserted into luciferase reporter vector as reporter plasmids: CCNG2-WT and CCNG2-MUT. The mimic NC and miR-1246 mimic were cotransfected with CCNG2 mRNA luciferase reporter plasmid to detect whether miR-1246 can bind to the 3′-UTR of CCNG2 mRNA. After 48 h of transfection, the cells were collected and lysed. Firefly and Renilla luciferase activity were measured using a Dual–Luciferase Reporter Assay System on a Glomax20/20 luminometer (Promega, Madison, WI, USA). Renilla served as an internal reference gene. The activation degree of the target reporter gene was calculated as the ratio of RLU of firefly to Renilla [32].

### Me-RIP sequencing

Total RNA was extracted by TRIzol from ovarian cancer tissues and cells and purified by PolyATtract mRNA isolation systems (A-Z5300, Aderr Tech., Beijing, China). Anti-m6A antibody (1:500, ab151230, Abcam), or anti-IgG antibody (ab109489, 1:100, Abcam) was added to IP buffer (20 mm Tris, pH = 7.5, 140 mM NaCl, 1% NP-40, and 2 mm EDTA) followed by incubation with protein A/G magnetic beads for 1 h for binding. IP buffer containing ribonuclease inhibitor and protease inhibitor was incubated with isolated and purified mRNA and magnetic bead–antibody overnight at 4°C. Afterward, the RNA was eluted with elution buffer and purified by phenol–chloroform. Eluted pri-miR-1246 was analyzed by RT-qPCR. The experiment was repeated three times [29].

### Xenograft mice model

A total of 36 female SPF BALB/C nude mice (5-week-old and weighing 18–22 g), purchased from Slake Experimental Animal Cooperation (Shanghai, China), were used for the tumorigenesis experiment. During the experiment, the mice were raised separately. ES2 ovarian cancer cells expressing oe-NC, oe-METTL3 + oe-NC, and oe-METTL3 + oe-CCNG2 constructed by lentivirus were applied in the subcutaneous tumorigenesis experiment of nude mice. The serum-free RPMI 1640 medium (Gibco) was used to resuspend ovarian cancer cells to a density of 1 × 10^6^ cells/200 μL. A total of 36 BALB/c nude mice were randomly divided into three groups with 12 mice in each group. The tumorigenesis experiment lasted for four weeks. After the nude mice were anesthetized with ether, they were routinely disinfected. The ovarian cancer cells were inoculated subcutaneously from the back of the right hind legs of each group of nude mice at a dose of 200 μL/mouse. All nude mice were kept singly housed in the same environment. The tumor size was measured every seven days and the length and width of the tumor were recorded. The tumor volume was calculated according to the volume = length × width^2^/2. After four weeks, the mice were euthanized and tumor tissues were dissected. The tumor size was measured, weighed, and photographed.

### Statistical analysis

Data statistical analysis was performed using SPSS 21.0 (IBM Corp., Armonk, NY, USA). Measurement data are expressed as mean ± standard deviation. Paired *t*-test was used to compare data between adjacent normal tissues with cancer tissues. Unpaired *t*-test was used for data comparison between the other two groups. One-way analysis of variance (ANOVA) was used for comparison between multiple groups. Repeated-measures ANOVA was used for statistical analysis in relation to time-based measurements within each group. The Kaplan–Meier method was adopted to calculate the survival rate of patients. Pearson’s correlation coefficient was used to analyze the correlation of the observed indicators. *p* < 0.05 means the difference is statistically significant.

## Supplementary information

Supplementary Table 1

Supplementary Table 2

Supplementary Table 3

Supplementary Table 4

Figure S1

Figure S2

Figure S3

Figure S4

Supplementary Figure Legends
