# Supplementary material for: METTL3 promotes the initiation and metastasis of ovarian cancer by inhibiting CCNG2 expression via promoting the maturation of pri-microRNA-1246
Source: Cell Death Discov. 2021 Sep 8;7:237. doi: 10.1038/s41420-021-00600-2 (PMC8426370; doi:10.1038/s41420-021-00600-2)
Supplement: Supplementary file 1 — Supplementary Table 1 [file 41420_2021_600_MOESM1_ESM.docx]

**Supplementary Table 1** Comprehensive score of GeneMANIA gene co-expression relationship

| Rank | Symbol | Score | Rank | Symbol | Score | Rank | Symbol | Score |
| --- | --- | --- | --- | --- | --- | --- | --- | --- |
| 1 | BSCL2 | 0.859101 | 21 | ST14 | 0.686655 | 41 | IGFBP2 | 0.651734 |
| 2 | BAIAP2L1 | 0.795832 | 22 | HOXB2 | 0.685895 | 42 | CLDN7 | 0.645551 |
| 3 | RHBDF2 | 0.781808 | 23 | KRT7 | 0.684034 | 43 | AXIN1 | 0.645302 |
| 4 | IP6K2 | 0.772445 | 24 | GALT | 0.683228 | 44 | MSLN | 0.644813 |
| 5 | MUC16 | 0.753995 | 25 | ESM1 | 0.678066 | 45 | EIF4G1 | 0.641744 |
| 6 | EPOR | 0.750674 | 26 | NFATC1 | 0.675169 | 46 | E2F5 | 0.639053 |
| 7 | RNF43 | 0.732802 | 27 | SLIT2 | 0.673587 | 47 | LAMC2 | 0.636701 |
| 8 | LGR5 | 0.728249 | 28 | ST6GAL1 | 0.673431 | 48 | ELF3 | 0.635211 |
| 9 | BBS9 | 0.721455 | 29 | PTGS1 | 0.672549 | 49 | SPP1 | 0.634954 |
| 10 | CD24 | 0.716694 | 30 | SMAD6 | 0.671542 | 50 | LAMB3 | 0.634188 |
| 11 | FOLR1 | 0.713866 | 31 | EPHA1 | 0.667463 | 51 | WFDC2 | 0.633338 |
| 12 | TACSTD2 | 0.713099 | 32 | HDAC6 | 0.66635 | 52 | CKB | 0.629773 |
| 13 | METTL3 | 0.710515 | 33 | FOXJ1 | 0.665744 | 53 | CLDN4 | 0.628137 |
| 14 | PRKX | 0.702195 | 34 | CEBPA | 0.665246 | 54 | NUMA1 | 0.625123 |
| 15 | SPINT1 | 0.696654 | 35 | TPD52 | 0.664089 | 55 | BCL2L1 | 0.624727 |
| 16 | PTP4A3 | 0.696624 | 36 | COL4A5 | 0.663562 | 56 | ABCA3 | 0.623125 |
| 17 | FCGR3A | 0.696454 | 37 | DSP | 0.659356 | 57 | ERBB2 | 0.622969 |
| 18 | SRC | 0.69405 | 38 | CLDN3 | 0.658215 | 58 | CP | 0.62177 |
| 19 | BAX | 0.691904 | 39 | NOTCH4 | 0.656036 | 59 | PRSS8 | 0.619408 |
| 20 | F2RL1 | 0.688876 | 40 | CX3CL1 | 0.653962 | 60 | LAMA5 | 0.61778 |
