# Supplementary material for: METTL3 promotes the initiation and metastasis of ovarian cancer by inhibiting CCNG2 expression via promoting the maturation of pri-microRNA-1246
Source: Cell Death Discov. 2021 Sep 8;7:237. doi: 10.1038/s41420-021-00600-2 (PMC8426370; doi:10.1038/s41420-021-00600-2)
Supplement: Supplementary file 2 — Supplementary Table 2 [file 41420_2021_600_MOESM2_ESM.docx]

**Supplementary Table 2** Patients’ clinicopathological features

| Clinicopathological feature | Case |
| --- | --- |
| Age at surgery (years) |  |
| ≤ 50.00 ^a^ | 22 |
| > 50.00 | 42 |
| Histological type |  |
| Serous | 43 |
| Mucinous | 9 |
| Other ^b^ | 12 |
| FIGO stage ^c^ |  |
| I | 8 |
| II | 17 |
| III | 27 |
| IV | 12 |

^a^ Mean age

^b^ Endometrioid, clear celland undifferentiated type

^c^ Revised staging for carcinoma adopted by the 2009 International Union of Obstetrics and Gynecology
