# Supplementary material for: METTL3 promotes the initiation and metastasis of ovarian cancer by inhibiting CCNG2 expression via promoting the maturation of pri-microRNA-1246
Source: Cell Death Discov. 2021 Sep 8;7:237. doi: 10.1038/s41420-021-00600-2 (PMC8426370; doi:10.1038/s41420-021-00600-2)
Supplement: Supplementary file 3 — Supplementary Table 3 [file 41420_2021_600_MOESM3_ESM.docx]

**Supplementary Table 3**Primer sequences for RT-qPCR

| Target | Sequence |
| --- | --- |
| METTL3-F | 5′-AAGCTGCACTTCAGACGAAT-3′ |
| METTL3-R | 5′-GGAATCACCTCCGACACTC-3′ |
| miR-1246-F | 5′-TGAAGTAGGACTGGGCAGAGA-3′ |
| miR-1246-R | 5′-TGAAGTAGGACTGGGCAGAGA-3′ |
| pri-miR-1246-F | 5′-TGAAGTAGGACTGGGCAGAGA-3′ |
| pri-miR-1246-R | 5′-TTTGGGTCAGGTGTCCACTC-3′ |
| CCNG2-F | 5′-TCCAAACTCAAAGAGACCAGC-3′ |
| CCNG2-R | 5′-TTCCACTTCAACTTCCCCAG-3′ |
| U6-F | 5′-CGAGCACAGAATCGCTTCA-3′ |
| U6-R | 5′-CTCGCTTCGGCAGCACATAT-3′ |
| GAPDH-F | 5′-TGGTCACCAGGGCTGCTT-3′ |
| GAPDH-R | 5′-AGCTTCCCGTTCTCAGCC-3′ |
