# Supplementary figures and images for: METTL3 promotes the initiation and metastasis of ovarian cancer by inhibiting CCNG2 expression via promoting the maturation of pri-microRNA-1246
Source: Cell Death Discov. 2021 Sep 8;7:237. doi: 10.1038/s41420-021-00600-2 (PMC8426370; doi:10.1038/s41420-021-00600-2)

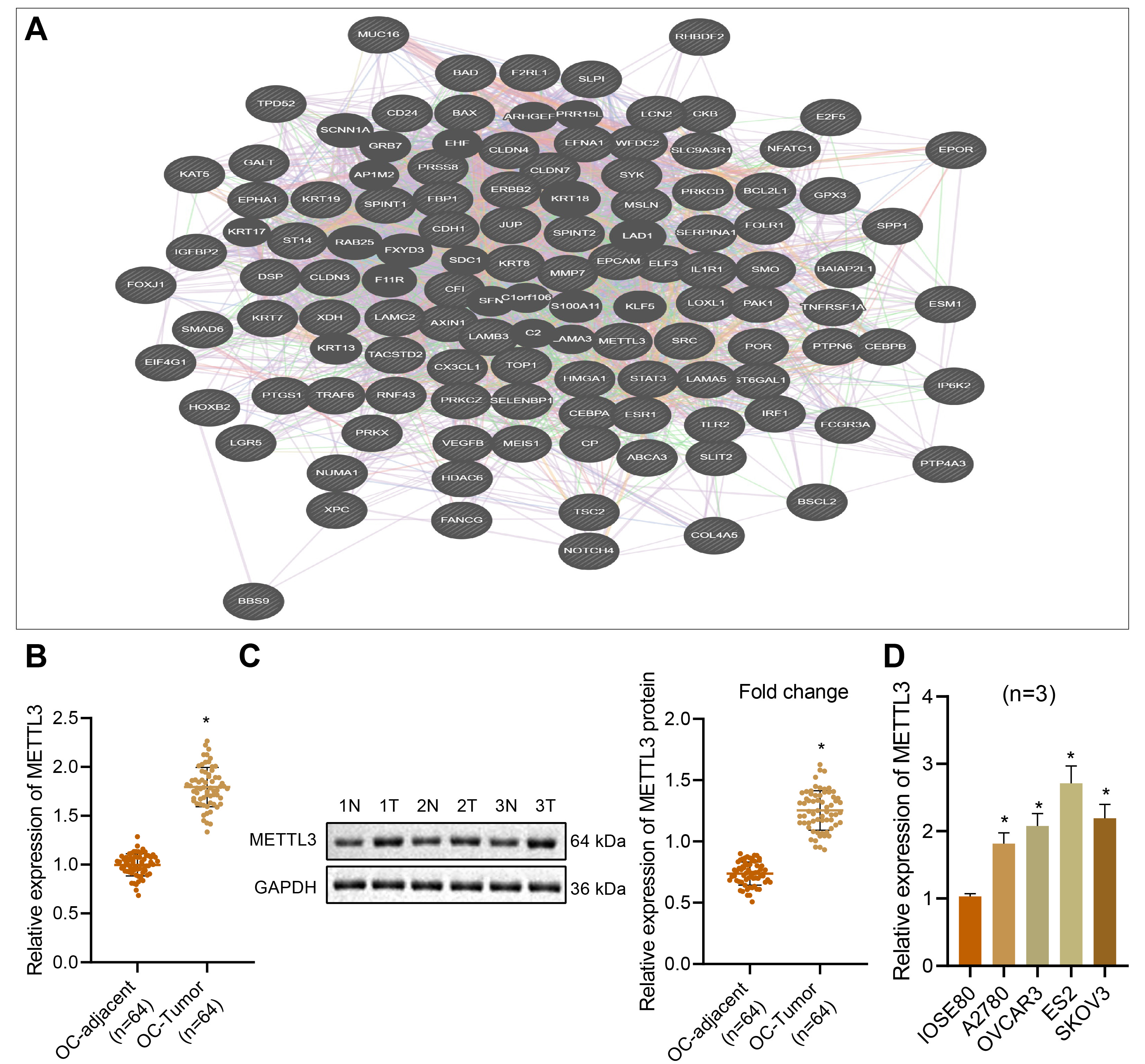

Supplement: Supplementary file 5 — Figure S1 [file 41420_2021_600_MOESM5_ESM.tif]

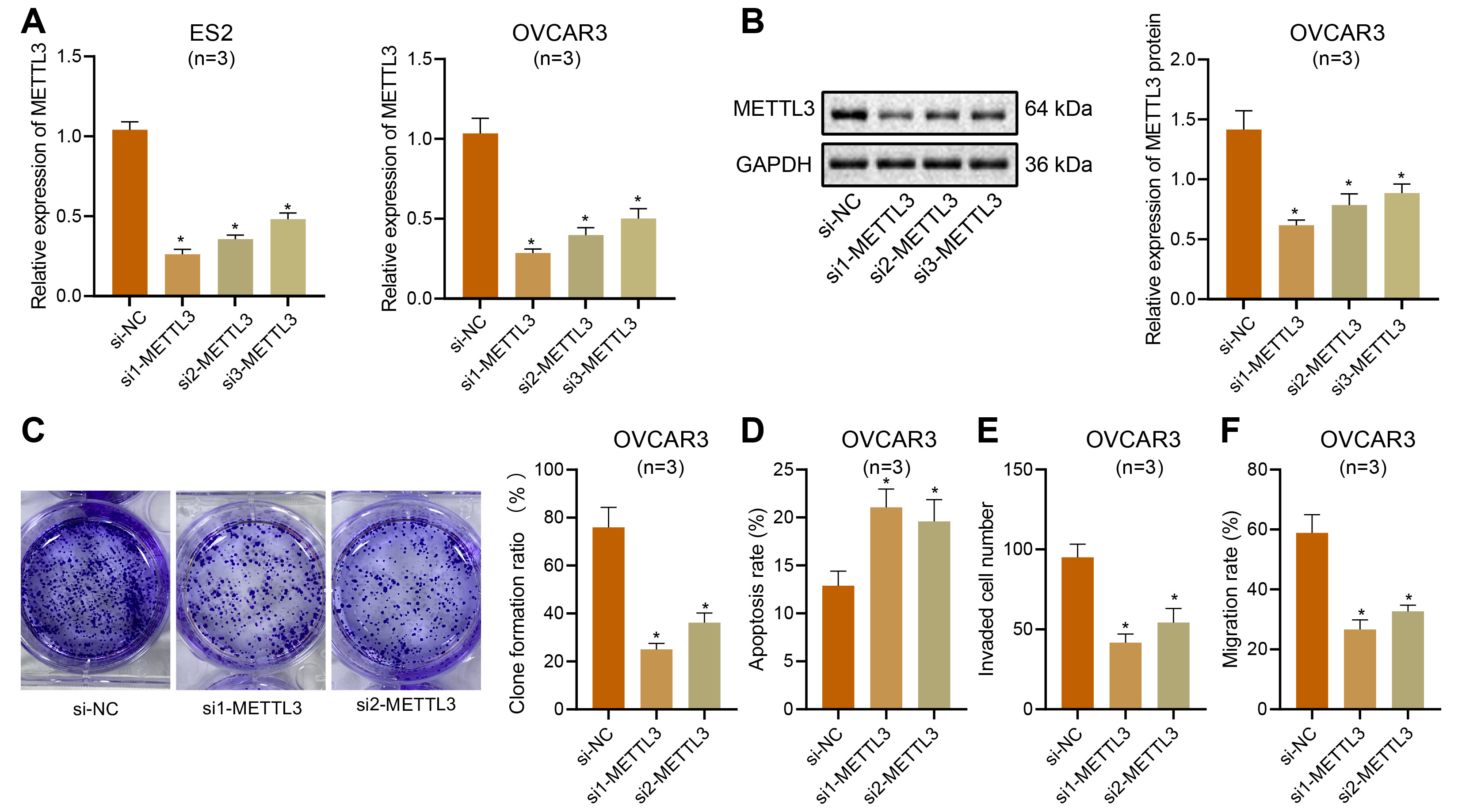

Supplement: Supplementary file 6 — Figure S2 [file 41420_2021_600_MOESM6_ESM.tif]

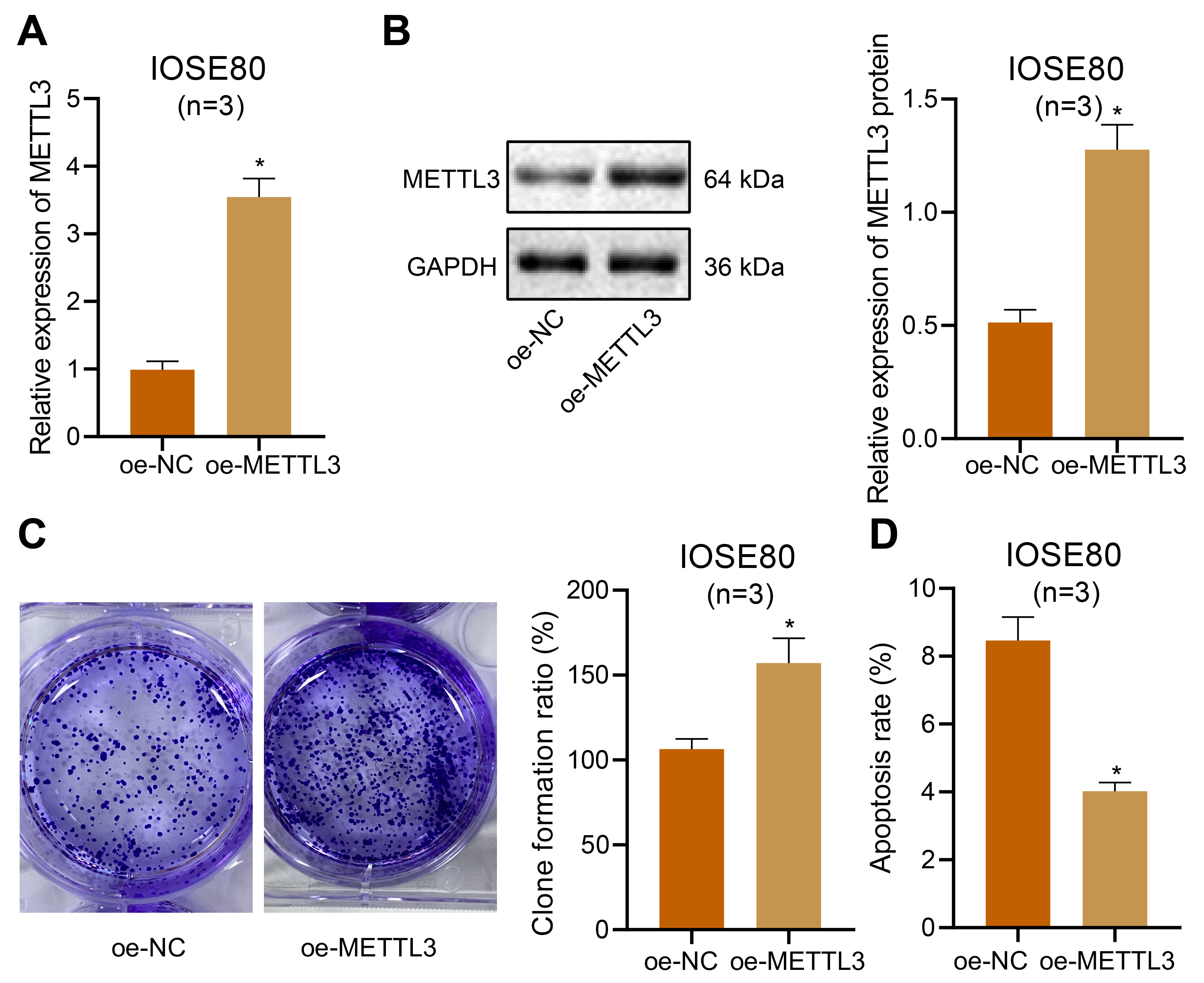

Supplement: Supplementary file 7 — Figure S3 [file 41420_2021_600_MOESM7_ESM.tif]

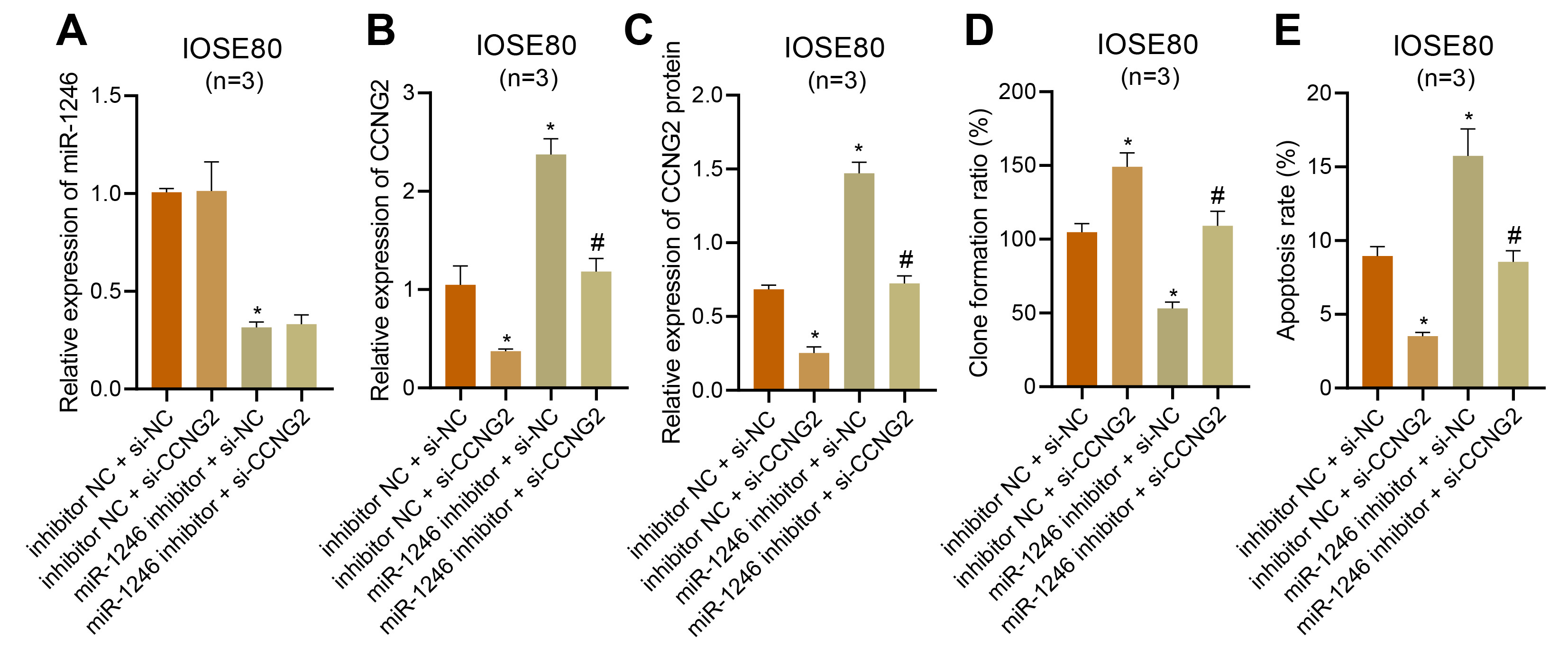

Supplement: Supplementary file 8 — Figure S4 [file 41420_2021_600_MOESM8_ESM.tif]
