# Supplementary material for: METTL3 promotes the initiation and metastasis of ovarian cancer by inhibiting CCNG2 expression via promoting the maturation of pri-microRNA-1246
Source: Cell Death Discov. 2021 Sep 8;7:237. doi: 10.1038/s41420-021-00600-2 (PMC8426370; doi:10.1038/s41420-021-00600-2)
Supplement: Supplementary file 9 — Supplementary Figure Legends [file 41420_2021_600_MOESM9_ESM.docx]

**Supplementary Fig. 1** METTL3 expression in ovarian cancer tissues and cells. A, Co-expression relationship network diagram of candidate genes. B, qPCR examining METTL3 expression of ovarian cancer (n = 64) and adjacent normal tissues (n = 64). C, Western blot examining METTL3 expression of ovarian cancer (n = 64) and adjacent normal tissues (n = 64). D, The expression of METTL3 detected by qPCR in IOSE80, A2780, OVCAR3, SKOV3, and ES2 cells. * *p* < 0.05, compared with adjacent normal tissues or IOSE80 cells. The experiments were repeated three times.

**Supplementary Fig. 2** METTL3 silencing inhibits the proliferation, migration and invasion of OVCAR3 cells and promotes their apoptosis. A, METTL3 expression in ES2 and OVCAR3 cells treated with si1-METTL3, si2-METTL3 or si3-METTL3 measured by qPCR. B, METTL3 protein expression in OVCAR3 cells treated with si1-METTL3, si2-METTL3 or si3-METTL3 measured by qPCR. C, Proliferation of OVCAR3 cells treated with si2-METTL3 or si3-METTL3 by colony formation test. D, Flow cytometric analysis of apoptosis in OVCAR3 cells treated with si2-METTL3 or si3-METTL3. E, Invasion of OVCAR3 cells treated with si2-METTL3 or si3-METTL3 measured by Transwell assay. F, Migration of OVCAR3 cells treated with si2-METTL3 or si3-METTL3 measured by scratch test. * *p* < 0.05, compared with the OVCAR3 cells treated with si-NC. The experiments were repeated three times with the most significant results presented.

**Supplementary Fig. 3** METTL3 overexpression promotes IOSE80 cell proliferation, migration and invasion, while inhibiting apoptosis. A, METTL3 expression in IOSE80 cells treated with oe-METTL3 tested by qPCR. B, METTL3 protein expression in IOSE80 cells treated with oe-METTL3 tested by Western blot; C, The proliferation of IOSE80 cells treated with oe-METTL3 detected by colony formation assay; D, Flow cytometric analysis of apoptosis of IOSE80 cells treated with oe-METTL3; * *p* < 0.05, compared with the IOSE80 cells treated with oe-NC. The experiments were repeated three times with the most significant results presented.

**Supplementary Fig. 4** miR-1246 silencing reduces proliferation and induces apoptosis of IOSE80 cells by upregulating CCNG2. IOSE80 cells were treated with miR-1246 inhibitor, si-CCNG2 or both. A, The expression of miR-1246 in IOSE80 cells. B-C, The expression of CCNG2 in IOSE80 cells examined by qPCR and Western blot. D, Proliferation of IOSE80 cells measured by colony formation assay. E, The apoptosis of IOSE80 cells measured by flow cytometry. * *p* < 0.05, compared with IOSE80 cells were treated with inhibitor NC + si-NC. # *p* < 0.05, compared with IOSE80 cells were treated with miR-1246 inhibitor + si-NC. The experiments were repeated three times with the most significant results presented.
